# Saffron improved depression and reduced homocysteine level in patients with major depression: A Randomized, double-blind study

**Published:** 2018

**Authors:** Gholamali Jelodar, Zahra Javid, Ali Sahraian, Sina Jelodar

**Affiliations:** 1 *Department of Physiology, Shiraz University, Shiraz,Iran*; 2 *Research Center for psychiatry and Behavior Science, Department of Psychiatry, School of Medicine, Shiraz University of Medical Sciences, Shiraz, Iran*; 3 *School of Medicine, Tehran University of Medical Sciences, Tehran, Iran*

**Keywords:** Saffron, Homocysteine, Major Depression, Crocus Sativus

## Abstract

**Objectives:**

A correlation between hyperhomocysteinemia, and depression has been reported. Saffron (*Crocus sativus*) is recommended for treatment of depression; hence, in this study the effect of co-administration of saffron and fluoxetine on plasma homocysteine and depression was evaluated.

**Material and methods:**

This was a 4-week randomized and double-blind clinical trial which was conducted from March 2013 to February 2014. In this trial, 40 male and females (20-55 years old) diagnosed with severe depression were selected and following filing the Beck form, were randomly divided into two groups. Experimental group was treated with fluoxetine 20 mg/day and saffron 30 mg /day and the control group received placebo and fluoxetine 20 mg/day for four weeks. Before treatment and at the end of the study, fasting blood samples were collected. For females, blood samples were collected on the third day of their menstrual cycle.

**Results:**

A significant reduction of homocysteine levels was observed in both sex in the experimental group compared to before treatment (p<0.04), while no such significant change was observed in the control group. A Beck questionnaire value showed lower level in both groups on the last day of treatment as compared to before treatment. There was no significant difference between the two groups in Beck value neither before nor after treatment.

**Conclusion:**

Saffron has beneficial effects on depression and homocysteine level in patients with major depression.

## Introduction

Worldwide, depression is a growing psychiatric disorder. Depressed people experience periods of reduced productivity and impaired social relationships (Allin et al., 2009 ▶, Nolen-Hoeksema and Girgus 1994 ▶). The exact mechanism, through which depression occurs, is not well known. Therefore, many arguments have been put forward. Currently, there is an interesting hypothesis which suggests the possibility of the presence of a genetic or metabolic basis for this disorder (Schildkraut, 1965 ▶). One of these metabolic changes is associated with homocysteine(Hcy)metabolism. Homocysteine is a nonessential amino acid produced following the metabolism of methionine, which has been associated with many diseases such as cardiovascular diseases and depression (Lentz 1997 ▶, Budge et al., 2002 ▶). It was reported that approximately 20–50% of patients with severe depression had increased total Hcy levels in the plasma (Fava, et al. 1997 ▶, Bottiglieri, et al. 2000 ▶). Furthermore, in a cross-sectional study, Tolmunen et al. determined that the subjects in the upper tertile for serum total Hcy had a more than two-fold higher risk of being depressed compared to the subjects in the lowest tertile for serum total Hcy (Tolmunen, et al. 2004 ▶). Another study confirmed that being in the lowest quartile of Hcy serum level was associated with fewer depressive symptoms after adjusting for sex, physical health, smoking, and other variables. A high level of Hcy correlates with depressive symptoms in community-dwelling middle-aged individuals (Sachdev et al. 2005 ▶). Therefore, as demonstrated by the above-mentioned epidemiological studies, an elevated Hcy concentration in plasma is very common in depression. Although the accumulation of Hcy has been implicated in the pathogenesis of depression (Plante 2005 ▶), whether Hcy is directly involved and acts as a primary cause of depressive symptoms is unclear (Bottiglieri 2005 ▶).

Excess amounts of homocysteine affects the nervous system through damaging the vascular system or destroying the neurons directly, either through inducing excitotoxicity or by development of oxidative stress (Koz et al., 2012 ▶ – Oldreive and Doherty 2007 ▶). Thereby, it causes different types of psychiatric disorders, such as depression, Alzheimer’s disease, schizophrenia and even Parkinson’s disease. 

During the last 6 decades, major targets of drugs used for treatment of depression, have been changed. The latest drugs used against depression belong to the selective serotonin reuptake inhibitors (SSRI) family and act more specifically on serotonin. Although these agents are very effective but due to undesirable side effects, they are also poorly compensated. Most of these drugs produce several adverse reactions, such as anticholinergic effects, orthostatic hypotension, arrhythmias and sexual dysfunction (Demyttenaere, 1997 ▶; MacDonald et al., 1997 ▶).

Saffron (*Crocus sativus*) is the world’s most expensive spice and apart from its traditional value as a food additive, recent studies have indicated its potential to be used as an anticancer agent and a memory enhancer as well as in the treatment of mild-to-moderate depression (Rios et al., 1996 ▶; Abe and Saito, 2000 ▶; Abdullaev, 2002 ▶ Noorbala, et al., 2005 ▶). The value of saffron (dried stigmas of *C. sativus*) is determined by the existence of three main secondary metabolites namely, crocin and its derivatives which are responsible for the color, picrocrocin which is responsible for the taste and safranal which is responsible for the odor. Indeed, saffron contains more than 150 volatile and aroma-yielding compounds. However, the exact mechanism of action of this herb on depression is not well known. Hence, this double-blind study was conducted to evaluate antidepressant effect of saffron, as well as its effect on homocysteine level as a cause of depression.

## Materials and Methods

This was a 4-week randomized and double-blind clinical trial conducted in the outpatient clinic of Hafez Psychiatric Hospital, Shiraz University of Medical Sciences, Shiraz, Iran from March 2013 to February 2014 with Clinical trial registration No. IRCT 2013 110915334N1.

**Patients**

Forty adult outpatients who met the diagnostic and Statistical Manual of Mental Disorders, fourth edition (DSM-IV) for major depression based on the structured clinical interview for DSM-IV and fulfilled the inclusion criteria participated in the trial. The inclusion criteria were being diagnosed with major depression; taking no antidepressant treatment during the last 6 months, signing the informed consent, and being between 18-55 years. In addition, patients were selected from both male and female genders. Patients were excluded from the study if the following items were positive: a history of suicide, presence of chronic diseases such as metabolic disease and cancer; having the ideas of committing suicide before or during the trial.

The trial was performed in accordance with the declaration of Helsinki and subsequent revisions and it was approved by Ethics Committee of Shiraz University of Medical Sciences and Psychiatric Research Center. Written informed consents were obtained from each patient before entering the study.

**Saffron capsule preparation**

Saffron used in this study was of high quality collected from Khorasan province, Iran. The stigmas of *C. sativus*, which are being used as a food additive and also a herbal medicine, was powdered and packed into capsules in a hygienic manner. Each capsule contained 30 mg of the powder. 

**Study design**

**Figure 1:**
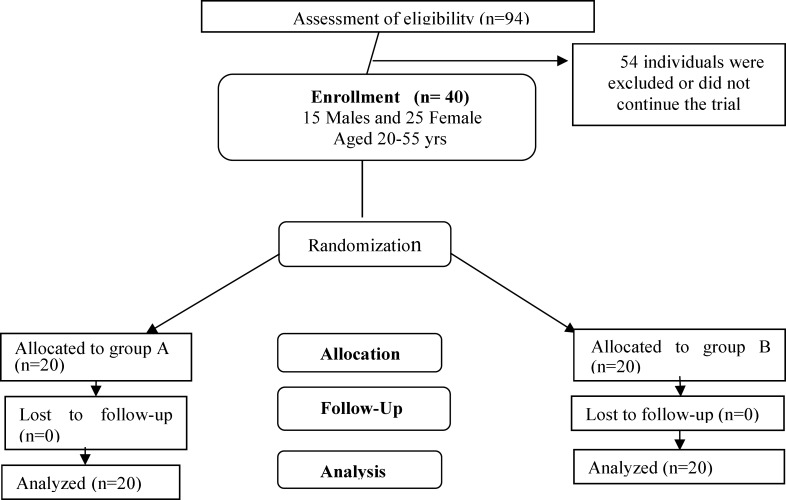
CONSORT flow diagram of the study.

A standard psychiatric interview was done by a specialist; then, a standard medical history was obtained and clinical evaluations were carried out on the patients who accepted to participate in the study. Each patient was asked to fill out a Beck rating score questionnaire, once on arrival at the clinic and once at the end of the 4^th^ week of treatment. Venous blood (5 ml) was obtained once before starting the treatment and once at the end of the 4^th^ week of treatment. The blood was then evaluated for total homocysteine. Patients were randomly divided into two groups in a 1:1 ratio using a computer-generated code (Figure. 1). The randomization was done by a third person who did not benefit from the study results and took charge of keeping the secrecy of data throughout the trial. The study group was named as “Group-A” and the control group as “Group-B” by the third person. Group-A received a capsule of saffron (30 mg) and a capsule of 20 mg fluoxetine, while Group-B (control group) received a capsule (which was similar to that of saffron) of placebo capsules and a capsule of 20 mg fluoxetine on a daily basis for 4 weeks. Throughout the study, the person who administrated the medications and the patients were blind to assignments. 

**Statistical analysis**

The Statistical Package for Social Science, SPSS for Windows, version 15.0 (SPSS, Chicago, IL) was used for data analysis. Paired t-test was used to compare results within the groups and independent t-test was used to compare results between the groups. Data are reported as means ± SEM. A two-sided p value less than 0.05 was considered statistically significant. Moreover, α and β powers were 0.05 and 80%, respectively.

## Results

**Figure 2:**
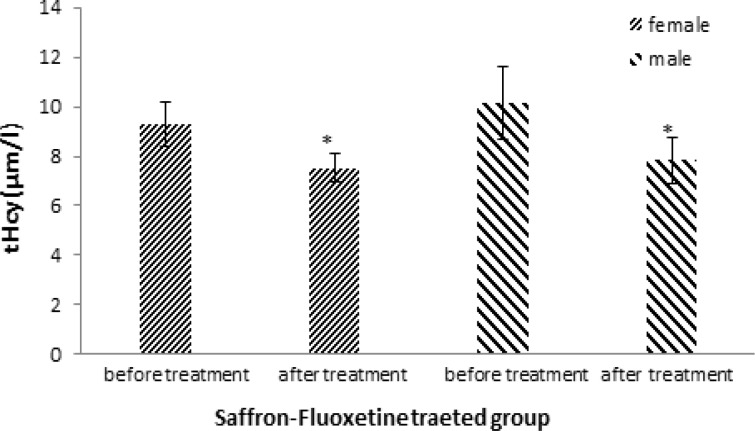
Comparison of total serum homocysteine( tHcy) before and after treatment in the experimental group (received saffron 30 mg plus fluoxetine 20 mg). Bars represent MEAN±SEM; * p<0.05

**Figure 3:**
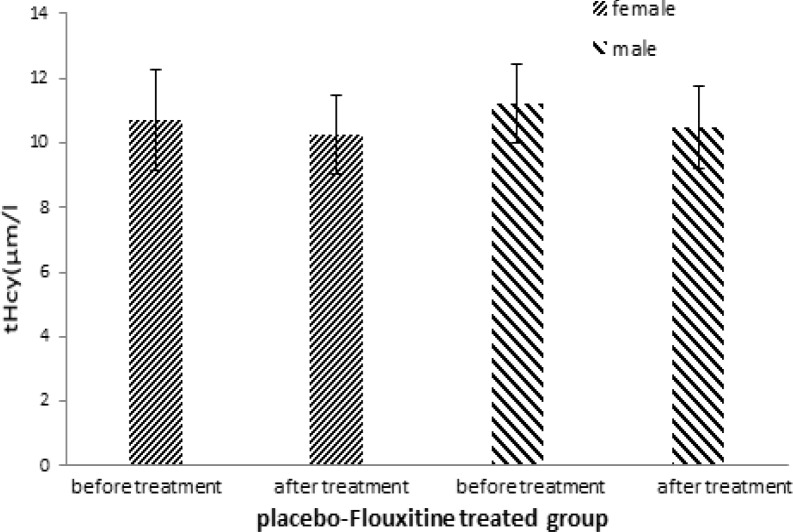
Comparison of total serum homocysteine(tHcy) before and after treatment in control group (received placebo plus fluoxetine 20 mg). Bars represent MEAN±SEM

In this trial, 40 patients including 25 women (65%) and 15 men (35%) were randomly allocated to two groups. Mean serum homocysteine concentration in experimental group was 9.31±0.9 µmol/l among women before treatment and 7.53±0.6 µmol/l after treatment which showed a significant decrease (p =0.040; Figure 2). Similarly, in men’s group there was a significant decrease in homocysteine level (10.14±1.45 µmol/l before *vs.* 7.83±0.94 µmol/l after treatment; p=0.049). The values within the control group were 10.71±1.55 µmol/l in women before and 10.25±1.24 µmol/l after treatment and in men’s group the values were 11.23±1.21 µmol/l before and 10.48±1.30 µmol/l after treatment (Figure 3). The changes in serum homocysteine concentration within the control group were not significant in both genders (p>0.05).

**Figure 4:**
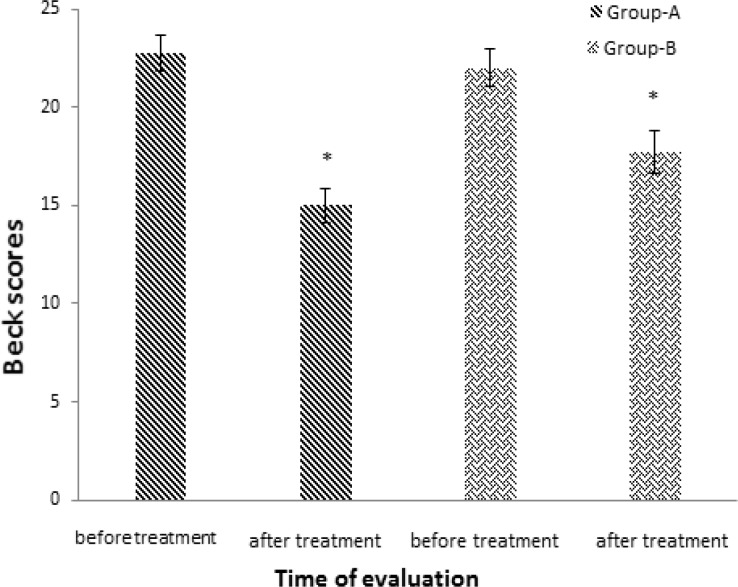
Scores of Beck questionnaire in both experimental (A) and control (B) groups. Bars represent MEAN±SEM; * p<0.05

**Figure 5:**
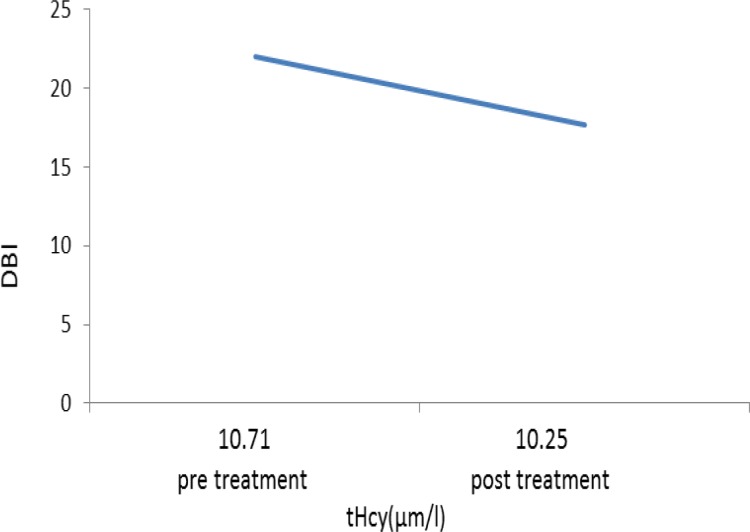
The relation between Beck score (DBI) and changes in homocysteine level in the control group

**Figure 6:**
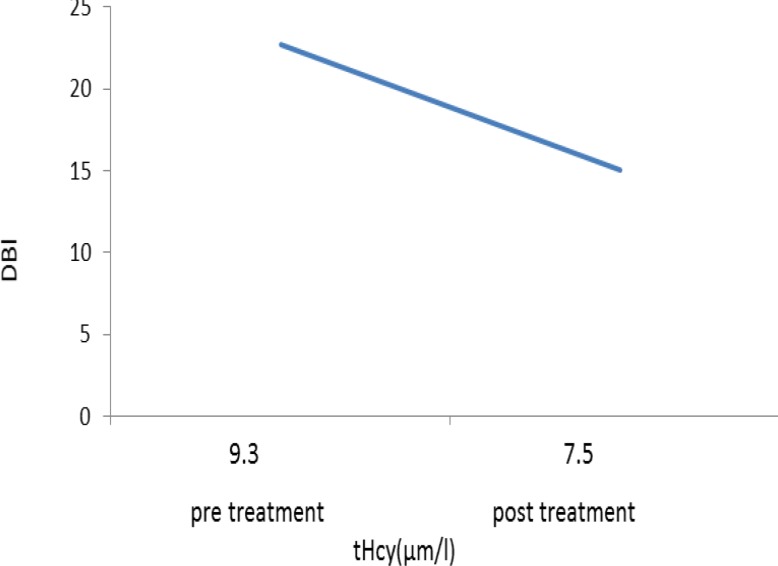
The relation between Beck score (DBI) and changes in homocysteine level in the experimental group.

A reduction in mean scores of Beck questionnaire was seen in both groups compared to before treatment (Figure 4) which was a sign of improvement in condition. However, the improvement in depression scale did not differ significantly between the two groups (p=0.056). Scores of Beck in experimental group was 22.73 ± 0.9 before treatment and 15.01 ± 0.87 after treatment which showed a significant decrease (p=0.003). Scores of Beck in control group was 22±0.98 before treatment and 17.73±1.09 after treatment which showed a significant reduction (p=0.008). The relation between homocysteine level and Beck score is presented in Figures 5 and 6.

## Discussion

Based on our data, administration of saffron did not potentiate the antidepressant effect of fluoxetine. Although there was no significant difference in Beck score between the two groups on the last day of the experiment, this score has lower value in the experimental group compared to the control group. Saffron significantly improved the level of homocysteine in both sex in the experimental group. Our results also showed that improvement of depression status is correlated with the decrease in serum homocysteine level. Higher levels of Hcy are associated with cell damage induced by oxidative stress and prompt many diseases including neurodegenerative disorders, depression and anxiety (Rinki et al., 2015 ▶)

Previous studies have also shown a correlation between increased levels of homocysteine and depression. Hyperhomocysteinemia (hHcy) were found more frequently in patients with major depression and hHcy may be considered as a useful clinical marker in patients with major depression as well as in PTSD patients (Topić, et all, 2010 ▶).

A correlation between depression and deficiency of folic acid, vitamin B12 and B6 has been reported. Deficiency of these vitamins results in an increase in homocysteine and is associated with deficiencies of neurotransmitters (serotonin, dopamine, noradrenaline, and γ-aminobutyric acid (GABA)) because folic acid, vitamin B12 and B6 are precursors to neurotransmitters. (Bottiglieri et al., 2000 ▶, Holford, 2003 ▶). It has been proposed that homocysteine exerts its effect via a glutamate receptor, called as N-methyl-D-aspartic acid receptor (NMDA) (Lipton et al 1997 ▶- Li J et al., 2013 ▶). This receptor is present on many cell types including hippocampal neurons and blood cells. Stimulation of NMDA receptor, leads to an influx of calcium and increase in reactive oxygen species (Waldmeier et al, 2000 ▶). Increases in homocysteine and glutamate synthesis in astrocytes of hippocampal matrix can either reduce the expression of NMDA receptors or increase their phosphorylated form. Alterations of these receptors have been reported to induce depression (Bukharaeva et al., 2015)

It has been reported that saffron has antagonistic activities on NMDA receptors (Hosseinzadeh et al., 2002 ▶ Lechtenberg et al., 2008 ▶). Saffron and its active constituent, crocin can prevent the impairment of learning and memory as well as the oxidative stress damage to the hippocampus induced by chronic stress. Thus, using these substances may alleviate cognitive deficits. Saffron affects neurotransmitters level such as norepinephrine, dopamine and serotonin by decreasing the activity of acetylcholine esterase and inhibiting the activity of monoamine oxidase in the brain (Khazdair et al., 2015 ▶). In our study, patients with severe depression, treated with *C. sativus* and flouxetine for 4 weeks, experienced significantly improved mood . These clinical findings were accompanied by the improvements in the Beck Depression Rating Scale results, in the both groups. Moreover, there were no significant differences between the two groups in terms of side effects. At least three clinical trials has reported that stigmas of *C. sativus* has antidepressant effects (Akhondzadeh et al., 2004 ▶, 2005; Noorbala et al., 2005 ▶). 

Nevertheless, this study is the first clinical trial that showed both antidepressant effects and serum homocysteine decreasing activity of saffron. The results of this study is in the line with a recent preclinical study that has reported an antidepressant effect (Akhondzadeh et al., 2004 ▶, 2005; Noorbala et al., 2005 ▶)..

Indeed, the results of this study indicate the efficacy of saffron in the treatment of depression. Also, a tolerable side-effects profile of saffron may suggest the safe application of *C. sativus* as a complementary treatment for depression.

## Compliance with Ethical Standards

Informed consent was obtained from all individual participants included in the study. This study was part of the Msc thesis of Miss Javid and was personally funded by the authors. 

## Conflict of interest

The authors declare no conflicts of interest.
